# Why Not Clean Milk?

**Published:** 1923-09

**Authors:** 


### Why Not Clean Milk?

SIR,—In the course of my holiday rambles in the North, I have come across dirty cows being milked in the dirtiest possible surroundings. I cannot too strongly express my horror to think that milk, probably to be seen, a few hours later, in brightly burnished churns on white tiles in some spotless city dairy shop, is being produced under unspeakably filthy conditions. I need not go into details which are only too familiar to anyone with eyes to see who wanders through some of our beautiful country districts.

Why can we not have clean milk, always and everywhere? I am not interested in all the complicated provisions of pasteurisation, Grade A, and the rest. Can you please tell me who is responsible for the perpetuation of the conditions to which I refer? I should be much obliged if you would enlighten me on the question. Who are the authorities responsible for seeing that milk is only taken from clean cows, milked with clean hands in clean cowsheds? And what are the precise powers of such authorities? Could you give me any indication why they do not use their powers? M. G. H.

[We purpose saying something on this subject shortly.—ED. THE HOSPITAL AND HEALTH REVIEW.]

---

### A Presentation Dental Clinic.

The offer of Major Astor, M.P., to build, and for two years to maintain, an adult dental clinic has been accepted by the Dover Town Council. It will provide the site, which will be near the infant welfare clinic.

